# Supplementary material for: Comparison of Methods for Incorporating Related Data When Developing Clinical Prediction Models: A Simulation Study
Source: Biom J. 2026 Aug 27;68(5):e70166. doi: 10.1002/bimj.70166 (PMC13520826; doi:10.1002/bimj.70166)
Supplement: Supplementary file 2 [file BIMJ-68-e70166-s001.zip › third submission code review - R2/reproducibility_supplement/results/Plots/one_plot_mean_cal_instability.pdf]

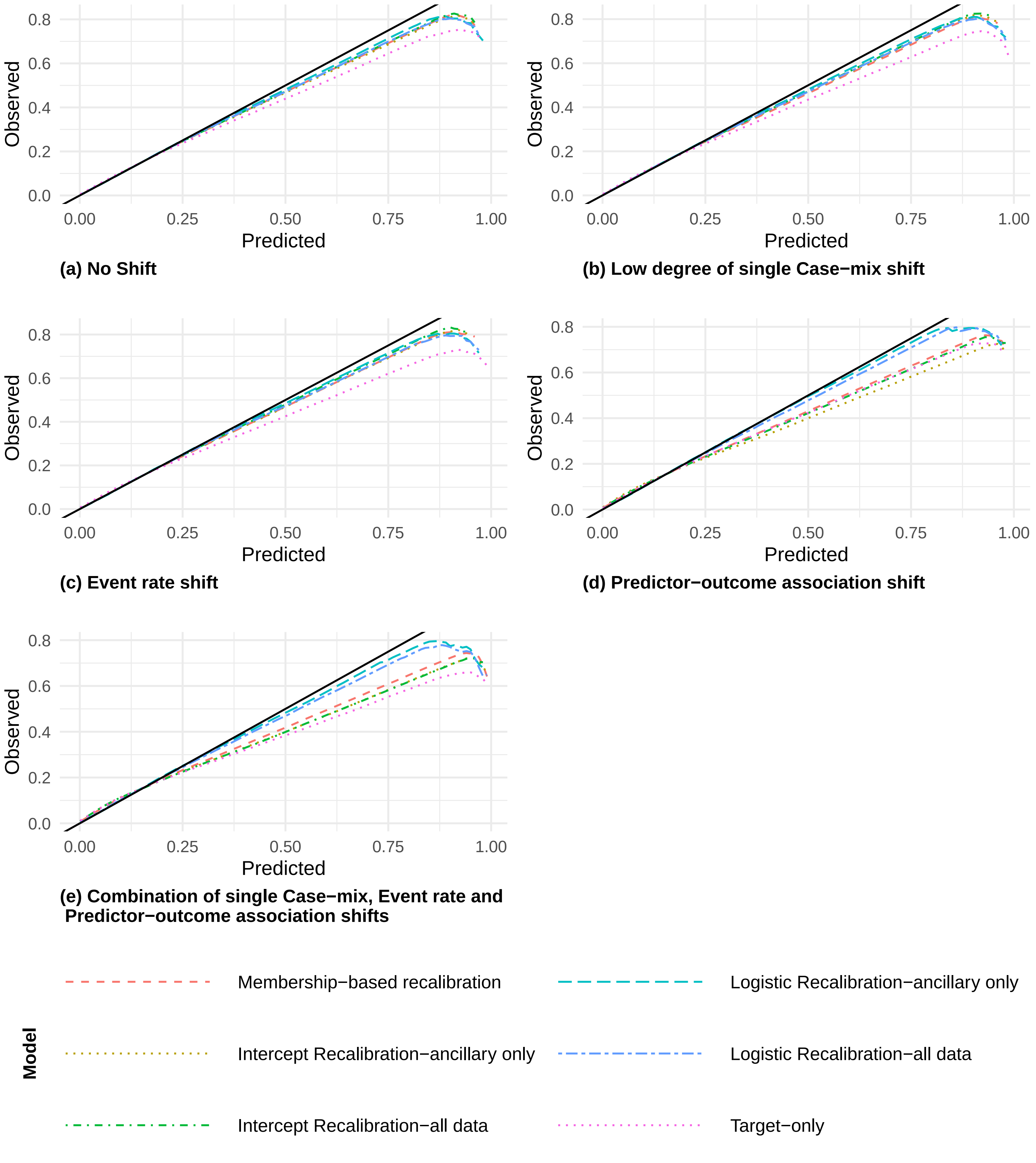
Observed
Predicted
0.0
0.2
0.4
0.6
0.8
0.00
0.25
0.50
0.75
1.00
(a) No Shift
(b) Low degree of single Case−mix shift
(c) Event rate shift
(d) Predictor−outcome association shift
(e) Combination of single Case−mix, Event rate and Predictor−outcome association shifts
Model
Membership−based recalibration
Intercept Recalibration−ancillary only
Intercept Recalibration−all data
Logistic Recalibration−ancillary only
Logistic Recalibration−all data
Target−only
